# Adverse drug events in pediatric intensive care are common, but improvement strategies exist and are effective

**DOI:** 10.1016/j.rppede.2016.07.002

**Published:** 2016

**Authors:** Karel Allegaert

**Affiliations:** a Intensive Care and Department of Surgery, Erasmus MC-Sophia Children's Hospital, Rotterdam, The Netherlands; b Department of Development and Regeneration, Katholieke Universiteit Leuven, Belgium

## Off label use is associated with adverse drug events

Pharmacotherapy is a powerful tool to improve outcome in children, but there is still an obvious need to reduce drug related problems through adverse drug event (ADE) prevention and management.^1^ An ADE can hereby be defined as ‘*any harmful and unintended event resulting from the use of a drug intended for diagnosis or therapy*'. Such an ADE definition is irrespective of the dose but more appropriate for children, since these patients still commonly receive off label drugs using doses extrapolated from adults. Besides the absence of labeling, (poly)pharmacy, inappropriate formulations, variability in dosing practices, difficulties to assess pharmacodynamics effects (e.g. sedation, pain), immaturity and organ dysfunction further raise the risk for ADEs in critically ill children.^2^^,^ 3 In pediatric and neonatal intensive care units (ICU), off label use of drugs is up to 80-90%, so significantly higher than the recently reported 30% in a primary pediatric care setting in Brazil.^4^ Unfortunately, these off label practices are associated by significant higher risks to develop ADE. Du et al. recently quantified this risk in a cohort of 697 consecutive PICU admissions.^5^ The overall risk was 13.1%, with an even higher risk after cardiovascular surgery, during infancy (<1 year of age), or in the setting of polypharmacy (≥6 drugs) or higher disease severity (Pediatric Risk of Mortality score upon admission).^5^

## Can we reduce the burden of adverse drug events?

Consequently, we need strategies to recognize and prevent ADEs in these populations, and a clinical pharmacy service (CPS) may be a very effective tool to do so. In this journal, Okumura et al. reported on their experience following introduction of a CPS into a single, 12 bed pediatric ICU.^6^ Already in a small sample of patients (n=53), the impact of the CPS on the detection of preventable ADE (n=141 events in 53 cases) was relevant. The most common interventions related to incompatibility of intravenous solutions (21%), or inadequate doses (17%) and-similar to other observations^1^^,^ 2 ^,^ 5-antimicrobial agents were overrepresented (5 of the top 10 drugs) in the intervention group.

This also means that such observations should guide secondary prevention programs to improve practices: *avoid the avoidance*, and *learn from your current practices*. CPS teams of clinical pharmacists should be an integrated part of multidisciplinary teams in addition to physicians, nurses and other health care providers. Clinical pharmacists should provide beside support in the prescription (dose, frequency) and the administration of drugs (e.g. incompatibilities). Because of the high ADE incidence, the ICU environment is an obvious choice.^6^

## Multidisciplinary teams to close the knowledge gap

As mentioned earlier, the currently still too limited knowledge on pharmacotherapy in ICU children is linked to a still too high incidence of ADEs. Multidisciplinary approaches-including clinical pharmacists-are very helpful to reduce preventable ADE. However, we should not underestimate the dynamics of such multidisciplinary teams once implemented. These groups can also generate new information on old drugs (*off label use should not remain off knowledge*) and they can develop and validate best practices and new dosing regimens, due to their expertise to collaborate bedside in the daily practice on pharmacotherapy: *let's make things better*.

## References

[1] Fabiano V, Mameli C, Zuccotti GV (2012). Adverse drug reactions in newborns, infants and toddlers: pediatric pharmacovigilance between present and future. Expert Opin Drug Saf.

[2] Allegaert K, Van den Anker J (2015). Neonatal drug therapy: the first frontier of therapeutics for children. Clin Pharmacol Ther.

[3] Allegaert K, Van den Anker JN (2015). Adverse drug reactions in neonates and infants: a population-tailored approach is needed. Br J Clin Pharmacol.

[4] Gonçalves MG, Heineck I (2016). Frequency of prescriptions of off-label drugs and drugs not approved for pediatric use in primary health care in a southern municipality of Brazil. Rev Paul Pediat.

[5] Du W, Tutag Lehr V, Caverly M, Kelm L, Reeves J, Lieh-Lai M (2013). Incidence and costs of adverse drug reactions in a tertiary care pediatric intensive care unit. J Clin Pharmacol.

[6] Okumura LM, da Silva DM, Comarella L Relation between safe use of medicines, off labels and clinical pharmacy services at pediatric intensive care. Rev Paul Pediatr.

